# *Saccharomyces boulardii* Modifies *Salmonella* Typhimurium Traffic and Host Immune Responses along the Intestinal Tract

**DOI:** 10.1371/journal.pone.0103069

**Published:** 2014-08-13

**Authors:** Rodolphe Pontier-Bres, Patrick Munro, Laurent Boyer, Rodolphe Anty, Véronique Imbert, Chloé Terciolo, Fréderic André, Patrick Rampal, Emmanuel Lemichez, Jean-François Peyron, Dorota Czerucka

**Affiliations:** 1 INSERM, U1065, Centre Méditerranéen de Médecine Moléculaire (C3M), Team 4 “Inflammation, Cancer, Cancer Stem Cells” Nice, France; 2 INSERM, U1065, Centre Méditerranéen de Médecine Moléculaire (C3M), Team 6 “Microbial toxins in host pathogen interactions” Nice, France; 3 INSERM, U1065, Centre Méditerranéen de Médecine Moléculaire (C3M), Team 8 “Hepatic complications in obesity” Nice, France; 4 Université de Nice-Sophia Antipolis, UFR Médecine, IFR50, Faculté de Médecine, Nice, France; 5 Centre Scientifique de Monaco, Monaco, Monaco; 6 CRO2 INSERM U911, Campus Santé Timone, Université Aix-Marseille, Marseille, France; Charité-University Medicine Berlin, Germany

## Abstract

*Salmonella enterica* serovar Typhimurium (ST) is an enteropathogenic Gram-negative bacterium that causes infection following oral ingestion. ST spreads rapidly along the gastrointestinal tract (GIT) and invades the intestinal epithelium to ultimately reach internal body organs. The probiotic yeast *Saccharomyces boulardii* BIOCODEX (*S.b*-B) is prescribed for prophylaxis of diarrheal infectious diseases. We previously showed that *S.b*-B prevents weight loss in ST-infected mice and significantly decreases bacterial translocation to the spleen and liver. This study was designed to investigate the effect of *S.b*-B on ST migration along the GIT and the impact of the yeast on the host's early innate immune responses. Bioluminescent imaging (BLI) was used to evaluate the effect of *S.b*-B on the progression of luminescent *Salmonella* Typhimurium (ST-*lux*) in the GIT of mice pretreated with streptomycin. Photonic emission (PE) was measured in GIT extracts (stomach, small intestine, cecum and colon) at various time periods post-infection (PI). PE analysis revealed that, 45 min PI, ST-*lux* had migrated slightly faster in the mice treated with *S.b*-B than in the untreated infected animals. At 90 min PI, ST-*lux* had reached the cecum in both groups of mice. Adhesion of ST to *S.b*-B was visualized in the intestines of the mice and probably accounts for (1) the faster elimination of ST-*lux* in the feces, and (2) reduced translocation of ST to the spleen and liver. In the early phase of infection, *S.b*-B also modifies the host's immune responses by (1) increasing IFN-γ gene expression and decreasing IL-10 gene expression in the small intestine, and (2) elevating both IFN-γ, and IL-10 mRNA levels in the cecum. BLI revealed that *S.b*-B modifies ST migration and the host immune response along the GIT. Study findings shed new light on the protective mechanisms of *S.b*-B during the early phase of *Salmonella* pathogenesis.

## Introduction

Gastrointestinal infections due to *Salmonella enterica* serovar Typhimurium are a major cause of diarrhea and mucosal inflammation and are responsible for severe systemic disease in both developing and industrial countries. Infection is usually due to the ingestion of contaminated food. Ingested *Salmonella* bacteria spread rapidly along the axis of the gastrointestinal tract (GIT) and penetrate the intestinal mucosa via three routes: (i) preferential invasion of specialized epithelial M cells situated in the dome region of Peyer's patches or overlying, solitary intestinal lymphoid tissues, (ii) active invasion of enterocytes, and (iii) through dendritic cells that intercalate epithelial cells by extending protrusions into the gut lumen (reviewed in [1]–[3]). If local host defenses are unable to limit the infection to the intestinal tract, the bacteria can spread systemically to the mesenteric lymph nodes, spleen and liver. The outcome of infection is determined by both bacterial and host factors, including the virulence of the infecting *Salmonella* spp, the ability of the host to mount an adequate inflammatory and immune response, and, ultimately, the host's ability to destroy the pathogen (reviewed in [4], [5]).

A lyophilized preparation of the probiotic yeast strain *Saccharomyces boulardii* (*S.b*-B) is used worldwide for the prevention and treatment of a variety of diarrheal diseases. *S.b*-B is currently the only probiotic yeast that has proven effective in controlled clinical trials (reviewed in [6]). *S.b*-B activities in the prevention of traveller's diarrhea have been investigated extensively. In a study of 1065 travellers who had visited various countries around the world, Kollaritsch et al. [7] reported that patients who received a placebo showed an incidence of diarrhea of 39.7% compared to 34.4% for patients who were given *S.b*-B at a dose of 250 mg/day (p = 0.019) and 28.7% for those who received *S.b*-B at a dose of 1 g/day (p<0.005).

Most cases of traveler's diarrhea (80–85%) are due to bacterial pathogens such as enterotoxigenic *Escherichia coli*, *Capylobacter jejuni*, *Shigella* species, and *Salmonella* species. *S.b*-B has been shown to have beneficial effects for the treatment of infectious diarrhea caused by *Salmonella, Shigella* and *E. coli* in animal models [8]–[12]. These effects are due to a number of direct mechanisms including binding to and elimination of pathogenic bacteria [9], [10], [13], actions on virulence factors [11], and modification of bacterial motility [14]. Indirectly, *S.b*-B also affect the host response, in particular through interference with the bacterial-induced signalling pathway [9], [10], [15], [16], [17] and beneficial cross-talk with the innate immune system [18]–[21].

Mice infected with *Salmonella enterica* serovar Typhimurium do not develop gastroenteritis but present typhoid fever-like symptoms. Several studies using this model have demonstrated that *S.b*-B can prevent ST translocation to the liver and spleen and has a protective effect on animal viability [9], [10]. Intestinal inflammatory responses can be induced during infection by serovar Typhimurium using mice pretreated with streptomycin [22]. Streptomycin causes transient clearance of the microbiota (>80%) and consequently disrupts colonization resistance for approximately 24–48 hours. This animal model is associated with high colonization levels in the cecum and colon, and the development of extensive intestinal inflammation within the first days after oral infection. The induction of analogous cytokine responses, and the involvement of identical virulence factors of *Salmonella* strongly suggest that mucosal inflammation in the streptomycin-pretreated mouse model, calves and humans is driven by similar pathogenic mechanisms [23], [24].

Bioluminescence imaging (BLI) is today a well-recognized technique for study of the establishment of infectious pathogens *in vivo* (reviewed in [25], [26]). Using ST transformed with the *lux* operon from *Photorhabdus luminescens*, Contag *et al.* [27] were the first to demonstrate the feasibility of detecting luminescence-generating microbes in a live mouse. BLI is a helpful means to monitor bacterial distribution, to distinguish between weak and strong virulent strains of *Salmonella*, to differentiate the susceptibility of mouse strains to infection, and to monitor antibiotic therapy.

We used the mouse model with streptomycin pre-treatment to investigate the effects of *S.b*-B on the kinetics of *Salmonella* migration in the gastrointestinal tract by BLI. For this purpose, the plasmid pSB417 containing the *lux*CDABE cassette from *Photorhabdus luminescens* was introduced into *Salmonella* strain SL1344 [28]. This allowed us to follow the migration of ST in the small intestine during the early phase of infection (0–90 min), up until the bacteria reached the cecum. Our study demonstrated that *S.b*-B modified the progression of *Salmonella* along the small intestine towards the cecum. *S.b*-B trapped the bacteria in the lumen, thereby favouring ST elimination in the feces and limiting translocation to the liver and spleen. *S.b*-B also modified the host immune response to the primary infection by increasing IFN-γ gene expression and reducing IL-10 gene expression in the small intestine. At 6 hours PI, upon ST arrival in the cecum, IFN-γ, and IL-10 mRNA levels were significantly increased in the yeast-treated group.

## Results

### *S.b*-B accelerates the progression of ST-*lux* along the GIT during the early stages of infection

**Figure 1:**
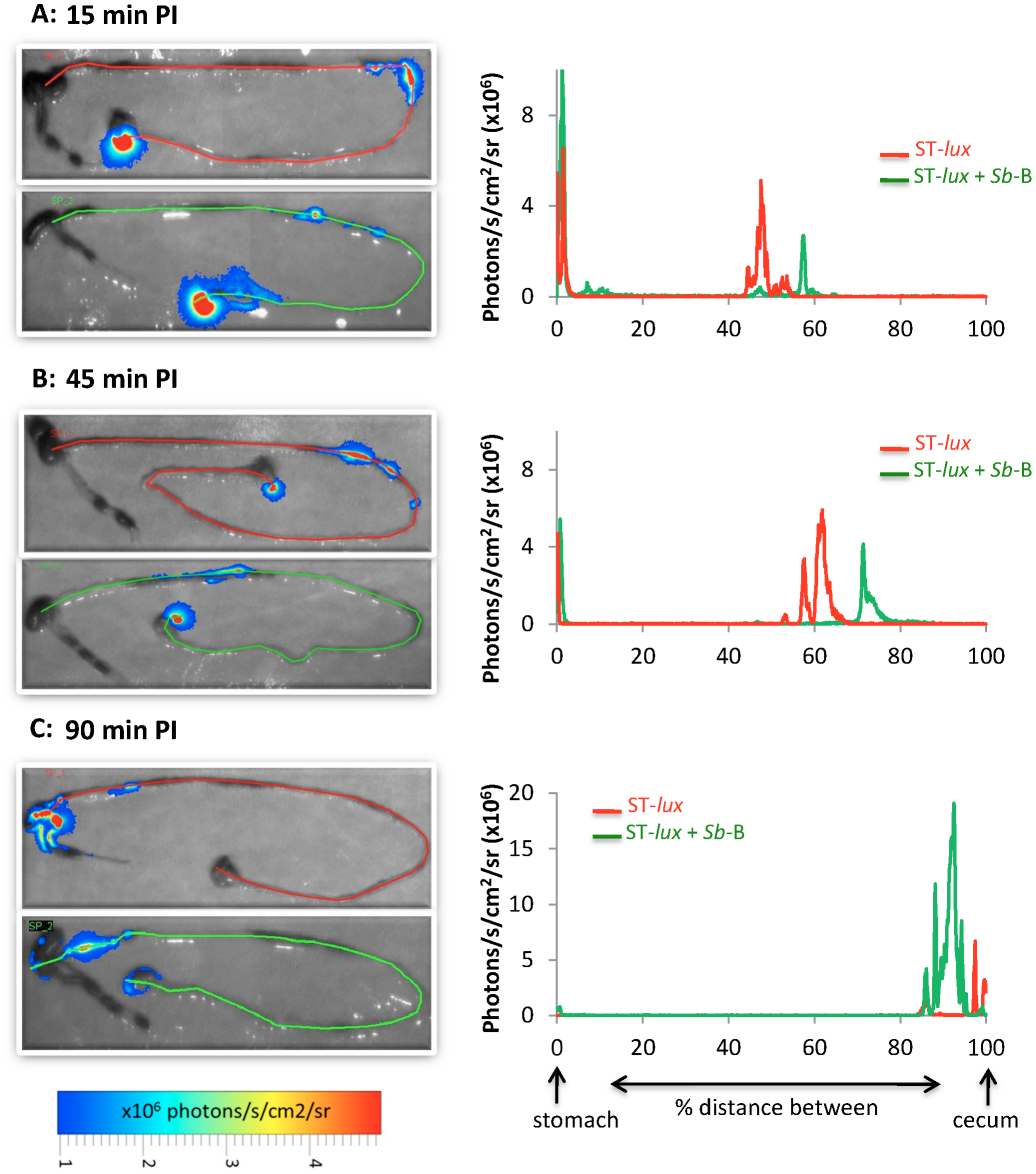
*In vivo* imaging of intestinal tracts extracted 15 min PI (A), 45 min PI (B), and 90 min PI (C) from mice infected with ST-*lux* alone (upper image, red line) or mice given both *S.b*-B and ST-*lux* (lower image, green line). Red and green lines were traced manually to measure the distance between the stomach and the cecum. Diagrams on the right represent the intensity of light along the distance between the stomach and cecum. Images were acquired with a Biospace Lab PhotonIMAGER and are displayed as pseudo-colour BLI peaks. Variations in colour represent light intensity at a given location: red represents the most intense light emission while blue corresponds to the weakest intensity (expressed as photon/second/cm^2^/steradian). Data are representative of 5 independent experiments.

Bioluminescent derivatives of ST strain SL1344 (ST-*lux*) were grown without shaking, and 10^8^ CFU were orally inoculated into streptomycin-pretreated mice, either alone or together with *S.b*-B. Infected mice were sacrificed at various times post-infection (15, 45 and 90 min PI) and the extracted intestinal tracts were analyzed by BLI (Fig. 1 A, B, and C). At 15 min-PI, the ST-*lux* administered alone had covered 50% of the distance between the stomach and the cecum. In the mice who had also received *S.b*-B, the bacteria had moved faster, covering 60% of the distance after the same amount of time (Fig. 1A). The difference in migration was even more pronounced at 45 min PI (Fig. 1B). In the mice given only ST-*lux*, the bacteria had covered 60% of the stomach/cecum distance, progressing at an average speed of 16 µm/sec, and formed three distinct peaks that probably reflected the presence of three different ST populations. In contrast, in the mice treated with *S.b*-B, the bacteria had moved faster (average speed 18 µm/sec) to cover 75% of the distance, and formed only one thin peak of light probably indicative of a single ST population. At 90 min PI (Fig. 1C), the bacteria had reached the cecum in both groups. Globally, photon emission was always greater in the mice treated with the yeast. Using a classical microbiological assay, we confirmed the kinetics of ST progression and demonstrated co-localization of viable populations of ST and *S.b*-B along the GIT (Tables S1 and S2).

The *Salmonella* burden was quantified in various gut sections by real-time PCR. For this purpose, GIT samples were obtained in parallel with BLI analysis (Figure S1A). Real-time PCR analysis allowed verification of the specificity of the ST 16S rRNA primer (Figure S1B).

**Figure 2:**
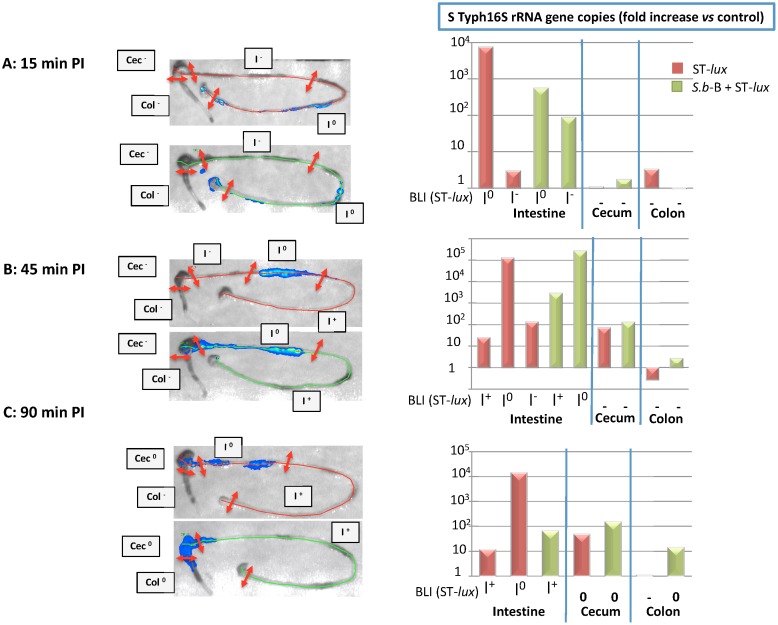
*In vivo* imaging of intestinal tracts extracted (A) 15 min PI, (B) 45 min PI, and (C) 90 min PI for mice infected with ST-*lux* alone (upper image, red line) and mice given both *S.b*-B and ST-*lux* (lower image, green line). The green and red lines were traced manually. Left panel: Sampling of the gut after the mice were imaged at various times PI (see Figure S2 for details). Right panel: Quantification of ST 16S rRNA gene copies by real-time PCR for each sample of GIT. Data are representative of 5 independent experiments.

At 15 min PI, no ST 16S rRNA gene copies were found in the cecum or colon tissues of the mice infected with ST-*lux* alone or those given both ST-*lux* and *S.b*-B (Fig. 2A). The highest levels of ST 16S rRNA gene copies were found in the small intestine samples exhibiting the maximal photon emission (I°) in both groups of mice. Interestingly, in *S.b*-B treated mice, a high ST population was detected in a portion of intestine without photon emission, noted “I^−^” At 45 min PI, equivalent amounts of ST 16S rRNA gene copies were detected in the small intestine and, a small quantity of ST genes was detected in the cecum of both groups (Fig. 2B). No bacteria were found in the colon 45 min PI (Fig. 2 B). At 90 min PI, ST 16S rRNA was detected in the small intestine and cecum of both groups. Bacteria were also detected in the colon of the *S.b*-B treated mice (Fig. 2 C).

ST 16S rRNA quantification confirmed that bacterial migration occurred faster in the *S.b*-B treated mice than in the animals who were given only ST. This approach allowed identification of small bacterial loads located before the bacterial peak detected by BLI. This bacterial population was probably not detected by BLI because its weak photon emission did not cross the tissue.

### *S.b*-B induces ST-*lux* elimination and ultimately decreases ST-*lux* translocation to the spleen and liver during the late stage of infection

**Figure 3:**
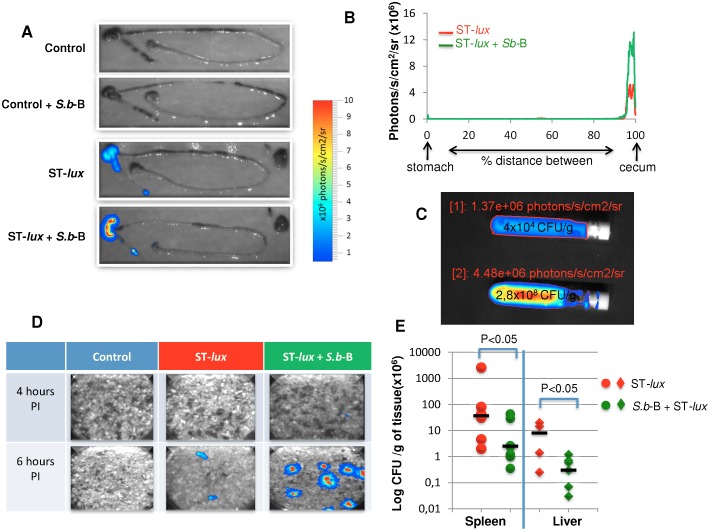
*In vivo* imaging of intestinal tracts extracted 6 hours PI from the control mice (A), mice treated by *S.b*-B alone, mice infected by ST-*lux* alone, and mice given both *S.b*-B and ST-*lux*. (B) Intensity of light along the distance between the stomach and the cecum. (C) Cecum samples isolated in tubes for BLI acquisition (areas of interest were delimited, and ROI were expressed as photon/second/cm^2^/steriadan) and CFU estimation after plating on agar medium. Upper tube: cecum from ST-*lux* alone infected mice; lower tube: cecum from *S.b*-B treated mice infected by ST-*lux*. (D) Snapshot of the cage environment 4 and 6 hours PI containing bacteria- contaminated feces from *S.b*-B challenged mice infected by ST-*lux*. (E) Bacterial counts evaluated 5 days PI in the liver and spleen. Data are representative of 5 independent experiments. The statistical significance of differences expressed as P values (P<0.05 was considered significant) are indicated above brackets.

We next monitored infection 6 hours PI, after ST had reached the cecum (Fig. 3A). Luminescence was localized in the cecum in both groups (Fig. 3A), but photon emission was more intense in the mice who had also been given *S.b*-B (Fig. 3A and B). Cecum samples of mice infected with ST-*lux* alone contained only 4×10^4^ CFU/g of tissue compared to 2.8×10^8^ CFU/g for the infected mice given *S.b*-B (Fig. 3C). These elevated ST levels are in agreement with the photon emission measurements (Fig. 3C) and were confirmed by ST 16S rRNA quantification (Figure S2). The animal cages were then examined to determine whether bacteria were eliminated in the feces. Beginning 6 hours PI, bioluminescent bacteria were detected in the feces of mice administered both ST-*lux* and *S.b*-B (Fig. 3D). No luminescent bacteria were found in the cages of control mice or mice infected with ST-*lux* alone. The absence of ST in the feces of mice given only ST-*lux* and the luminescence signal observed in the cecum suggested that *Salmonella* could have translocated to internal organs, i.e. the liver and spleen. This prompted us to examine these organs for the presence of ST in both groups of mice. As hypothesized, ST counts in the liver and spleen were higher in the mice infected with ST only, indicating greater ST translocation from the intestines (Fig. 3E). Reduced ST translocation to the liver and spleen as the result of *S.b*-B treatment was reflected by greater ST excretion in the feces.

### *S.b*-B binds ST and modifies ST distribution in the GIT

**Figure 4:**
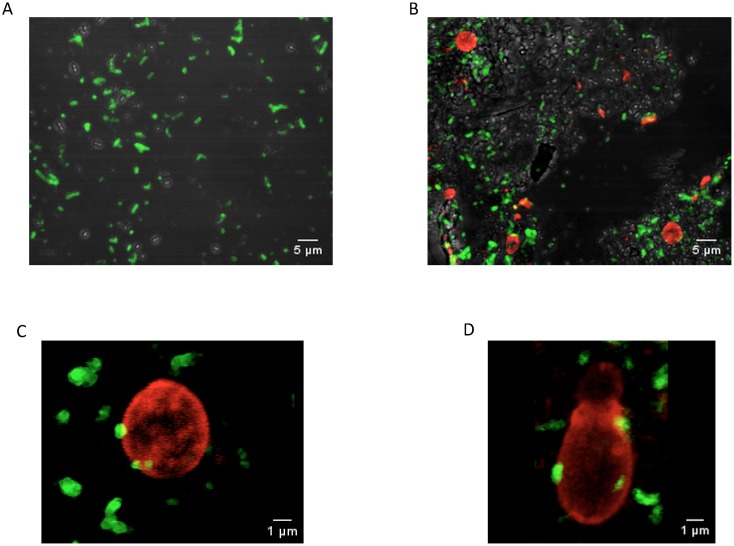
Immunohistological localization by confocal microscopy of FITC–labelled ST (green) and TRITC-labelled *S.b*-B (red) isolated in the intestine of mice 45 min PI: (A) intestinal lumen of ST-only infected mice, (B) intestinal lumen of *S.b*-B and ST treated mice. (C) and (D) adhesion of ST (green) to the *S.b*-B cell wall (red).

Adhesion of bacteria to the yeast has been previously documented both *in vitro* [9], [13] and *in vivo* [10]. Using specific antibodies that recognize ST or *S.b*-B, the distribution of ST in the portion of intestine exhibiting maximal BLI (I°) was analyzed by confocal microscopy at 45 min PI. ST was found to be distributed regularly throughout the intestinal lumen in the mice given ST alone (Fig. 4A and Video S1). The presence of *S.b*-B modified the ST distribution in the lumen: ST tended to cluster around *S.b*-B (Fig. 4 B and Video S2). 3D analysis revealed an interaction between the yeast and ST (Fig. 4 C, D and Video S3). Interestingly, budding yeasts were observed (Fig. 4D and Video S4), indicating that live *S.b*-B can survive in the intestinal lumen. Together, these results support the hypothesis that adhesion of ST to the yeast cell wall modifies ST distribution in the lumen, thereby improving ST elimination in the feces.

### *S.b*-B modifies innate immune responses induced by ST in the small intestine

As IL-10 and IFN-γ responses have been shown to play a key role in *Salmonella* clearance during infection, the levels of transcription of these cytokines were evaluated along the GIT. Expression of these cytokines was not modified in (i) the non-infected control mice, and (ii) in mice following streptomycin and/or *S.b*-B treatment (Figure S3). In infected mice, levels of mRNA coding for these cytokines were not modified in the colon during the 6 hours after ST-*lux* administration (Figure S4).

**Figure 5:**
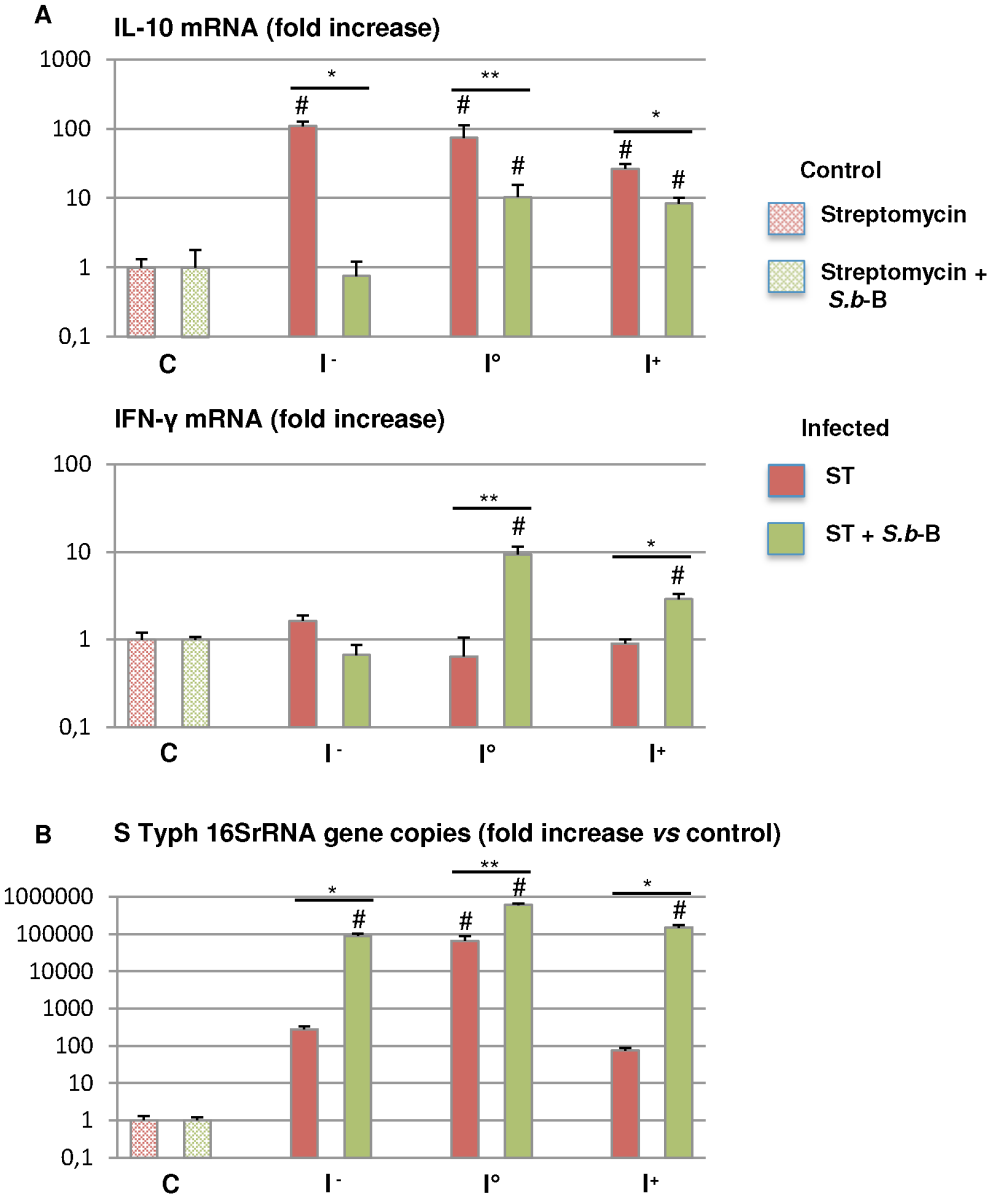
Cytokine expression measured by real-time PCR in the intestine of mice infected by ST-*lux* alone and mice challenged with *S.b*-B (A). Following BLI measurement, small intestine samples were obtained: I^−^ without photon emission, I° maximal photon emission, I^+^ after bacterial passage (see Fig S2 for more details). Data are presented as relative mRNA (fold change ×10^2^). The number of ST 16SrRNA gene copies in each sample is shown in (B). N = 5, # p<0.05 treated animals *vs* controls, *<0.05 ST alone *vs* ST+ *S.b*-B, **<0.02 ST alone *vs* ST+ *S.b*-B.

We then compared cytokine expression in the small intestine and cecum of both groups of mice infected with luminescent *Salmonella*, i.e. the group that had received *S.b*-B and the group that had not been given the yeast. In a first step, we sought to determine whether ST migration significantly modifies IFN-γ and IL-10 expression in the various portions (I^−^, I°, I^+^) of the small intestine (Fig. 5A). IL-10 expression was significantly up-regulated along the small intestine of the ST infected mice (p<0.05 compared to control mice) (Fig. 5A). This up-regulation occurred very early in the I^−^ portion of the intestine that presented low ST burdens compared to the I° portion (Fig. 5B). IL-10 gene expression was significantly decreased along the intestine in the ST infected mice that were treated with *S.b*-B (p<0.05 *vs* mice infected with ST alone). In contrast, IFN-γ gene expression was not modified in ST alone infected mice, but it was significantly up-regulated in the intestine upon bacterial arrival (I° and I^+^) in the mice infected and treated with *S.b*-B (p<0.02 for I° and p<0.05 for I^+^ *vs* mice infected with ST alone) (Fig. 5A). Modulation of IL-10 and IFN-γ expression was independent of the higher bacterial load observed in the different portions of the intestine of mice given *S.b*-B compared to the mice infected only by ST (Fig. 5B).

These findings demonstrate that ST induced IL-10 expression very soon after inoculation, even in that portion of small intestine exposed to just a very small bacterial population. In mice given both *S.b*-B and ST, the IL-10 level was significantly decreased and the level of IFN-γ was significantly up-regulated in the small intestine.

### *S.b*-B modifies ST-induced cytokine expression in the cecum

**Figure 6:**
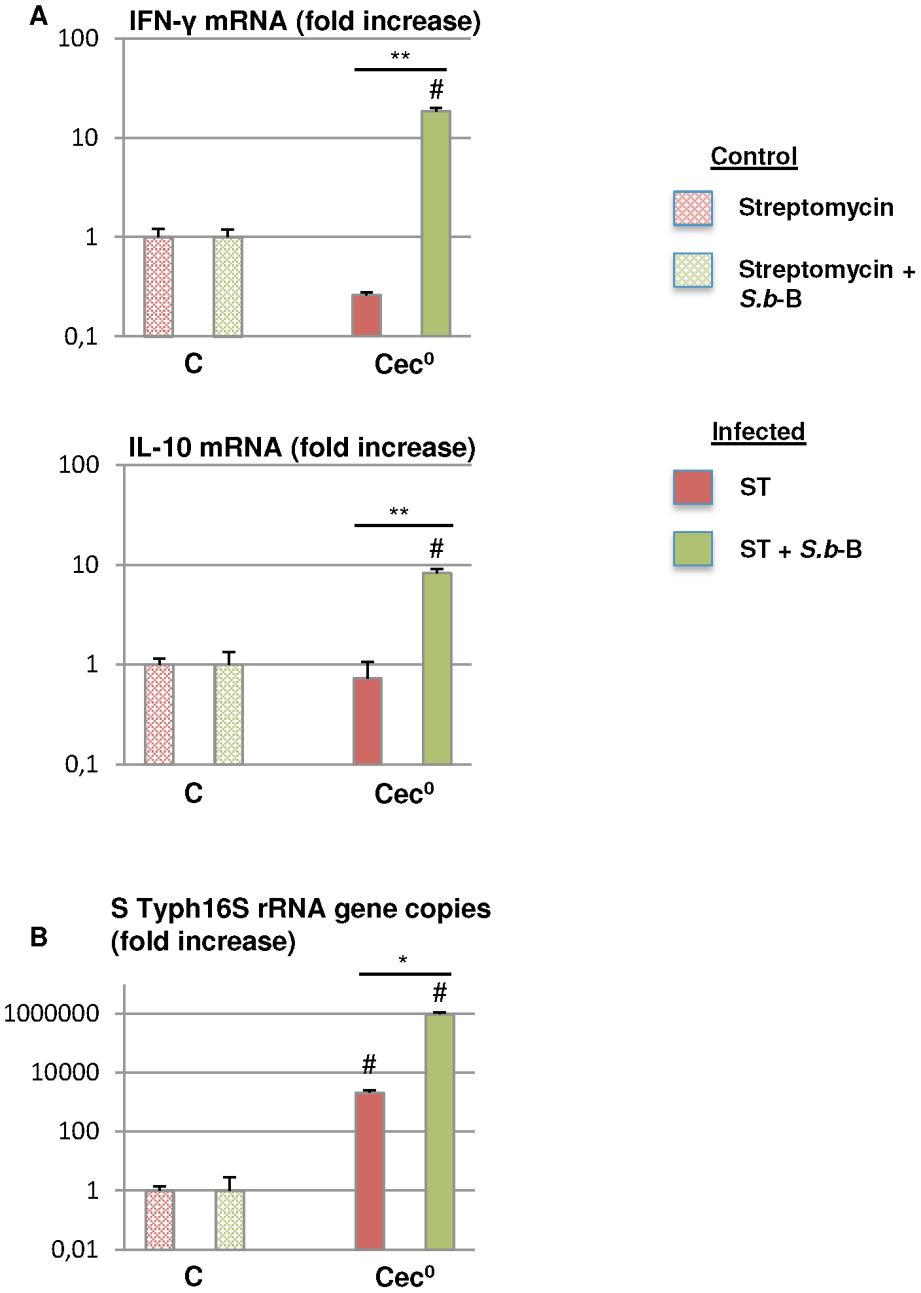
IFN-γ and IL-10 gene expression measured by real-time PCR 6 hours PI in the cecum (Cec°) of mice infected by ST-*lux* alone and mice challenged with *S.b*-B (A). Data are presented as the relative mRNA (fold change ×10^2^). The number of ST 16SrRNA gene copies in each sample is shown in (B). N = 5, # p<0.05 treated animals *vs* controls, *<0.05 ST alone *vs* ST+ *S.b*-B, **<0.02 ST alone *vs* ST+ *S.b*-B.

Determination of IFN-γ, and IL-10 gene levels in the cecum (Fig. 6A) revealed that ST did not significantly modify expression in this tissue (Fig. 6 A). Up-regulation of the expression of both IFN-γ and IL-10 cytokines was observed at 6 hours PI in the mice given *S.b*-B (p<0.02 compared to the mice given ST alone) (Fig. 6A). These variations were independent of the higher bacterial load observed in the cecum of the mice administered *S.b*-B compared to animals only given ST (Fig. 6B).

Overall, these results demonstrate that, 6 hours PI, IFN-γ and IL-10 gene expression levels in *S.b*-B treated mice were significantly up-regulated in the cecum.

## Discussion

Infections due to *Salmonella enterica* serovars represent a serious health problem worldwide. Although *Salmonella* infections can be treated with antibiotics, these drugs are often not readily available in endemic areas, and antibiotic resistance is on the rise. Vaccines exist for typhoid fever, but they afford only moderate protection in adults and are ineffective in children. Furthermore, no vaccines are currently available for nontyphoid *Salmonella* serovars. New and improved strategies are thus needed to treat *Salmonella* infections.

To our knowledge this is the first study that investigate the *in vivo* effects of the probiotic yeast *S.b*-B on ST infection during the early stages after inoculation. This is achieved by using a bioluminescent imaging (BLI) technique. As shown by our data, BLI is extremely useful for monitoring ST infections of the GIT. Using this technique, we found that *S.b*-B modifies ST migration kinetics. ST progressed at an average speed of 16 µm/sec, in agreement with earlier measurements in the gut lumen of streptomycin-treated mice [29]. In our study, *S.b*-B was found to accelerate the speed of ST-*lux* progression to an average of 18 µm/sec. In the mice infected with ST-*lux* alone, the bacteria migrated in several groups, visualized as several BLI peaks. In contrast, in the animals given both *S.b*-B and ST-*lux*, ST-*lux* progressed as a single, more homogenous population, visualized as a single BLI peak. Adhesion of ST to the yeast is a possible explanation for the formation of a single peak. This hypothesis is supported by data demonstrating that *S.b*-B progression along the intestinal tract followed ST progression (Table S2). Furthermore, 3D analysis of confocal microscopy images revealed ST adherent to *S.b-B* and the presence of ST clusters around yeasts. To our knowledge it is the first study demonstrating adhesion of ST to living budding yeast. One hypothesis is that yeast-adherent bacteria were trapped in the gut lumen, and moved faster than the non-adherent bacteria that were moving along the villi and crypts of the intestinal epithelium. The presence of yeast-adherent bacteria also implies a reduction in the number of ST that can adhere to the intestinal epithelium and translocate to other tissues. This hypothesis is supported by the drop in ST translocation to the spleen and liver observed in *S.b*-B treated mice.

Our study clearly shows that the level of cytokine expression can vary between small intestine and the cecum of ST-infected mice. When mice were given both ST and *S.b*-B, we observed the up-regulation of IFN-γ and the down-regulation of IL-10 in the early phase of infection (0–90 min) along the small intestine, even in the portion containing very small loads of bacteria. These results favor the hypothesis that *S.b*-B acts as an immune-modulator which protects ST-infected animals. These results also correlate with the up-regulation of other pro-inflammatory cytokines in the cecum (Figure S5). This pro-inflammatory priming response exerted by *S.b*-B in the early phase of infection is beneficial for host survival and prevents loss of body weight during the late phase of infection (Figure S6). As recently reported by other authors, inflammation and infection initiate the recruitment of mononuclear phagocytes that are required for host survival [30]. Interestingly, IL-10 expression is later up-regulated in the cecum. After 6 hours of infection, the increase of IL-10 may signal the beginning of a “return to normal” and down-modulation of the immune response. The differences in cytokine expression observed in the intestine and cecum concur with recent data indicating that the mechanism of invasion of the cecal mucosa is distinct from the extension-mediated transepithelial transport described for ileal immune cells [31], [32].

IFN-γ produced by activated T cells and natural killer (NK) cells has been shown to play an important role in the host defense against intracellular pathogens such as ST [33]. Systemic administration of this cytokine during the first few days after challenge with *Salmonella* reduces the severity of infection in mice [34]. Interestingly, *S.b*-B treatment induced up-regulation of IFN-γ gene expression in both the small intestine and the cecum, indicating a protective role of *S.b*-B through stimulation of the innate immune response along the GIT. IFN-γ and IL-10 are two cytokines that have antagonistic effects on macrophages: IFN-γ stimulates macrophage activity in the presence of infection, whereas IL-10 has inhibitory effects. Interestingly, *S.b*-B induced up-regulation of IFN-γ and down-regulation of IL-10 in the same portion of the small intestine where the maximal ST population was observed. It would be of a major interest to determine the immune effector cells targeted by *S.b*-B that are responsible for the production of IFN-γ and IL10 which induce a protective effect against ST infection.

Altogether, our study demonstrates that BLI permits: (i) visualization of *Salmonella* migration following oral administration, and (ii) investigation of the dynamic behavior of the immune response in the gut during the early period PI. Our results suggest that *S.b*-B adhesion to *Salmonella* was involved in the modification of ST migration and triggered fecal elimination of the bacteria along with decreased bacterial translocation. Moreover, *S.b*-B clearly exerts a strong effect on the innate immune responses, as revealed by the up-regulation of IFN-γ and down-regulation of IL-10 along the GIT. Further investigations are necessary to identify the cell populations targeted by *S.b*-B at different sites along the gut.

## Materials and Methods

### Microorganisms

The virulent, streptomycin-resistant *Salmonella enterica* serovar Typhimurium SL1344 (ST) strain [14] used in this study was kindly provided by Stéphane Meresse, Centre d'Immunologie de Marseille-Luminy, CNRS-INSERM Université de la Méditerranée, Marseille, France. Luminescent ST (ST-*lux*) were obtained after transformation by electroporation of the plasmid pSB417 containing the *lux*CDABE cassette from *Photorhabdus luminescens* [28]. Bacteria were stored in Luria-Bertani (LB) medium plus 15% glycerol at −80°C and grown in LB broth overnight at 37°C without shaking. *S. boulardii* (*S.b*-B) cultures were obtained by inoculating a commercial lyophilized preparation of the yeast (Ultra-Levure, BIOCODEX, France) that was grown overnight at 37°C, with shaking, in Halvorston minimal medium containing 2% glucose, as previously described [15].

### Mouse experiments

Female, 6 to 8-week old C57BL/6 mice from specific pathogen-free stocks were purchased from Harlan (Harlan, France). During the course of these studies, sentinel animals were screened for common murine pathogens every 2 months. All animals were housed in individual, HEPA-filtered cages with sterile bedding and had free access to sterilized water and food. All animal experiments were performed in accordance with the Animals Scientific Procedures Act (1986) and were approved by the Institutional Ethics Committee on Laboratory Animals (CIEPAL-Azur, Nice Sophia-Antipolis, France) (PEA Number: NCE/2013-68).

### Mouse *Salmonella* infection model

Bioluminescent *Salmonella* (ST-*lux*) were grown in LB medium with ampicillin at 37°C without shaking up until the late exponential phase, then washed twice with PBS and re-suspended in PBS. CFU were determined by plating serial dilutions of cultures on LB-agar medium containing ampicillin. Infection was obtained using a recently described enteritis model [22]. In brief, mice were deprived of food and water for four hours prior to administration of 20 µg of streptomycin/mouse by oral gavage. After two hours, food and water were provided *ad libidum*. Twenty-four hours after oral streptomycin treatment, food and water were once again withdrawn for four hours, after which 10^8^ live ST-*lux* bacteria in 200 µl of PBS were administered by oral gavage. Mice challenged with *S.b*-B received 10^7^ CFU of yeast in 200 µl of PBS 48 hours before infection, 24 hours before infection (at the same time as streptomycin treatment), and during infection with ST-*lux*. Control mice were given 200 µl of PBS. As summarized in Figure S7, the mice were divided into the following groups: non-treated (10 mice), streptomycin-treated (5 mice), streptomycin- and *Sb*-B treated (5 mice), streptomycin- and ST-*lux* treated (10 mice) and streptomycin-, *S.b*-B- and ST-*lux* treated (10 mice).

### Imaging of bioluminescence from animals and organs

Images were acquired using the Biospace Lab PhotonIMAGER (Biospace Lab, Paris, France) according to the manufacturer's instructions. Acquisition and analysis were performed using Biospace Lab Photo acquisition 2.8 and M3 Vision software, respectively. For *ex vivo* bioluminescent imaging of organs, mice were euthanized by cervical dislocation at designated time points, and the entire intestinal tract was extracted. Image acquisitions were matched to photonic signals integrated for 5 min. BLI signals were quantified from selected regions of interest (ROI) and expressed as photon/second/cm^2^/steradian (ph/s/cm^2^/Sr). For the comparison of signals (a) among the different mice, and (b) among the intestinal tracts of different mice, background photon emission was subtracted and the signals were presented on the same scale.

### Quantification of ST and *S.b*-B in the intestinal tract of infected mice

After bioluminescent imaging of the intestinal tracts, the portions of interest were recovered, weighed and then homogenized in 2 ml of sterile PBS using Ultra-Turrax T25 (Fisher Scientific, France). Viable counts were performed by spotting 100 µl of serial dilution onto MacConkey agar plates containing ampicillin for *Salmonella* determination and onto YEPD-agar for *S.b*-B determination.

### Translocation of ST to the spleen and liver

Five days after infection, the mice were sacrificed and dissected. The spleen and liver were recovered, weighed and then homogenized in 2 ml of sterile PBS using Ultra-Turrax T25. Viable counts were performed by spotting 100 µl of serial dilution onto LB-agar medium containing ampicillin for *Salmonella* determination.

### Real-time PCR

For ST quantification and analysis of modifications in cytokine gene expression, tissue samples were collected, immediately snap-frozen in liquid nitrogen at the site of surgery and stored at −80°C until processing. Snap-frozen tissues were homogenized with Precellys CK14 beads in a Precellys 24 tissue homogenizer/grinder (Ozyme, France). RNA was extracted from the samples with a NucleoSpin RNA/Protein kit (Macherey-Nagel, France) and quantified by spectrophotometry (Nanodrop ND1000). Next, 1 µg of RNA from each sample was reverse transcribed using a Revert Aid First-Strand cDNA Synthesis Kit (Promega, France) according to the manufacturer's instructions. The cDNA reaction mixture was diluted 1/10, and used for each real-time reaction. Real-time analysis was performed using SYBR Green (Applied Biosystems) and a 7900HT Fast Real-Time PCR system. The data were analyzed using a comparative threshold cycle (Ct) method (Applied Biosystems). Relative gene expression units were calculated by normalization: the Ct values for the 36B4 gene were subtracted from the Ct values of the target gene. The relative quantifications were also calculated by determining the difference between the treated mice and the controls. The genes analyzed in our study and the respective primers are listed in Table S3.

### Immunofluorescence microscopy

45 min PI, ST-*lux* mice and *S.b*-B + ST-*lux* treated mice were sacrificed. The GITs were extracted and the location of the maximal ST-*lux* loads was determined using a Biospace PhotonIMAGER, as described hereafter. The intestine samples exhibiting maximal photon emission were embedded in Cryomatrix compound (Thermo Shandon), snap-frozen in liquid nitrogen, and stored at −80°C. Cryosections (5 µm) were air-dried for 1 hour at room temperature, fixed in acetone (10 min at −20°C), washed, then blocked with normal goat and swine serum for 1 hour. The sections were immunostained overnight at +4°C with a polyclonal goat isothiocyanate (FITC)-labeled antibody to *Salmonella* (KPL, USA). *S.b*-B cells were detected using polyclonal rabbit antibody (obtained in our laboratory after immunization of rabbits with an active fraction of *S.b*-B) following staining for 2 hours at room temperature with a swine TRITC-conjugated anti-rabbit antibody (DakoCytomation, Denmark). Sections were mounted with Mowiol (Sigma, France) and dried at room temperature overnight in the dark. Image acquisition and analysis were performed at the C3M Imaging Core Facility. Optical fields showing bacteria and yeast in the lumen or in the proximity of intestinal mucosa were selected for analysis. Samples were examined using a Zeiss LSM 510 Meta confocal microscope mounted on an Axiovert 200M microscope stand with a Zeiss Plan-Apochromat 63×/1.4 oil objective. Images (512×512 format) were collected using 405, 488, 543 and 633 nm laser lines for excitation and appropriate emission filters. Pinholes were set to 1 Airy Unit (AU) for each channel. Z-stacks were acquired with an optimized interval and acquisition parameters were optimized for the brightest planes of the stack. Plane image and 3D reconstructions were obtained using ImageJ software.

### Statistical analysis

All experiments were repeated at least five times. Results are presented as the mean ± the standard error of the mean (SEM). Statistical significance of the various gene expressions was determined first by ANOVA, then with Student's t-test for each group of interest. A level of *P*<0.05 was considered significant.

## Supporting Information

Figure S1 **Gut sampling and primer validation for ST 16S rRNA.** Panel A presents the results of gut sampling after the mice were imaged at different times post infection. During the early phase of infection (15 and 45 min), maximum photon emission was localized in the intestine. Three intestine samples were therefore obtained, corresponding to: the site of maximum photon emission reflecting the maximal bacterial concentration (“I°”), the ileum, which showed no photon emission or bacteria (“I^−^”), and the duodenum, which had already been in contact with the bacteria (“I^+^”). At 15 min, the cecum and colon did not exhibit any photon emission, and were noted “cec^−^” and “col^−^”, respectively. At 45 min PI, the cecum presented photon emission and was noted “cec°”. At 90 min PI, photon emission was observed in both the cecum (“cec°”) and the colon (“col°). No photon emission was seen in the intestine, but the entire intestine had been in contact with ST and is noted “I^+^”. Panel B presents the primer validation for ST 16S rRNA. Several controls were performed. First, we verified that the primer did not recognize other bacteria present in the intestinal microbiota. RNA was extracted from the intestine of normal mice and mice treated by streptomycin. No copies of ST16S rRNA were amplified in these samples. No copies were found in the intestine of mice treated by streptomycin and *S.b*-B. In contrast, our primer recognized an ST 16S rRNA copy in RNA extracted from ST culture. When the same quantity of ST culture was mixed with a sample of uninfected intestine (I^−^), we found the same quantity of ST16S rRNA copies, demonstrating the absence of interference when intestinal tissue was added.
(PPTX)

Figure S2 ***In vivo*** **imaging of the intestinal tract extracted 6 hours PI from control mice, mice treated by** ***S.b*** **-B alone, mice given ST-** ***lux*** **alone, and mice administered both** ***S.b*** **-B and ST-** ***lux*** **(A).** Diagrams represent the intensity of light along the distance between the stomach and cecum. (B) Quantification of 16S rRNA in various parts of the GIT from mice infected by ST-*lux* alone and mice treated with *S.b*-B during infection.
(PPTX)

Figure S3 **IFN-γ and IL-10 gene expression measured by real-time PCR in the samples of intestine, cecum and colon from control mice (blue bars), and mice treated by streptomycin alone or with** ***S.b*** **-B.**
(PPTX)

Figure S4 **IFN-γ (A) and IL-10 (B) gene expression measured by real-time PCR in the different samples of intestine, cecum and colon obtained from mice infected by ST-** ***lux*** **alone and mice mice treated with** ***S.b*** **-B and infected for different periods of time (15, 45, 90 min and 6 hours).** Empty bars: controls. PE: Photon Emission. Annotation of PE: “O” maximal PE, “−” no PE, “+” PE after ST passage. Data are representative of 5 independent experiments.
(PPTX)

Figure S5 **GM-CSF, IL-1 and TNF-α gene expression measured by real-time PCR 6 hours PI in the cecum from mice infected by ST-** ***lux*** **alone and infected mice treated with** ***S.b*** **-B.**
(PPTX)

Figure S6 ***S.b*** **-B treatment inhibits wasting disease due to ST-infection.** Survival was monitored every day up until 6 days PI (A). Weight loss on day 5 PI is expressed as a % compared to the initial weight of the mice before infection (B).
(PPTX)

Figure S7 **Experimental protocol.**
(PPTX)

Table S1 **Mean concentration (CFU×10^6^/g of tissue) of** ***Salmonella*** **detected along the intestinal tract after oral inoculation of mice.** Different portions of the intestinal tract were removed, weighed and homogenized for plating of serial dilutions onto McConkey agar plates, as described in Material and Methods. Data are expressed as CFU×10^6^/g of tissue. ND: not determined. N = 5.
(DOCX)

Table S2 **Mean concentration (CFU×10^5^/g of tissue) of** ***S.b*** **-B detected along the intestinal tract after oral inoculation of mice.** Different portions of the intestinal tract were removed, weighed and homogenized for plating of serial dilutions onto YEPD agar plates, as described in Material and Methods. Data are expressed as CFU×10^5^/g of tissue. ND: not determined. N = 5.
(DOCX)

Table S3 **Primers used in this study.**
(DOC)

Video S1 **3D reconstruction of the intestinal lumen of mice infected with ST alone.** Intestine sample exhibiting maximal photon emission (I°) 45 min PI. ST were labelled with anti-ST-FITC antibody.
(AVI)

Video S2 **3D reconstruction of the intestinal lumen of** ***S.b*** **-B treated and ST-infected mice.** Sample of intestine exhibiting maximal photon emission (I°) 45 min PI. ST were labelled with anti-ST-FITC antibody. *S.b*-B were identified with anti-*S.b*-B antibody followed by incubation with a secondary TRITC-antibody.
(AVI)

Video S3 **3D reconstruction of ST (FITC) around** ***S.b*** **-B (TRITC) in the sample of intestine exhibiting maximal photon emission (I°) 45 min PI.**
(AVI)

Video S4 **3D reconstruction of ST (FITC) around** ***S.b*** **-B (TRITC) in the sample of intestine exhibiting maximal photon emission (I°) 45 min PI.**
(AVI)
