# Supplementary material for: Saccharomyces boulardii Modifies Salmonella Typhimurium Traffic and Host Immune Responses along the Intestinal Tract
Source: PLoS One. 2014 Aug 13;9(8):e103069. doi: 10.1371/journal.pone.0103069 (PMC4145484; doi:10.1371/journal.pone.0103069)
Supplement: Figure S1 — Gut sampling and primer validation for ST 16S rRNA. Panel A presents the results of gut sampling after the mice were imaged at different times post infection. During the early phase of infection (15 and 45 min), maximum photon emission was localized in the intestine. Three intestine samples were therefore obtained, corresponding to: the site of maximum photon emission reflecting the maximal bacterial concentration (“I°”), the ileum, which showed no photon emission or bacteria (“I−”), and the duodenum, which had already been in contact with the bacteria (“I+”). At 15 min, the cecum and colon did not exhibit any photon emission, and were noted “cec−” and “col−”, respectively. At 45 min PI, the cecum presented photon emission and was noted “cec°”. At 90 min PI, photon emission was observed in both the cecum (“cec°”) and the colon (“col°). No photon emission was seen in the intestine, but the entire intestine had been in contact with ST and is noted “I+”. Panel B presents the primer validation for ST 16S rRNA. Several controls were performed. First, we verified that the primer did not recognize other bacteria present in the intestinal microbiota. RNA was extracted from the intestine of normal mice and mice treated by streptomycin. No copies of ST16S rRNA were amplified in these samples. No copies were found in the intestine of mice treated by streptomycin and S.b-B. In contrast, our primer recognized an ST 16S rRNA copy in RNA extracted from ST culture. When the same quantity of ST culture was mixed with a sample of uninfected intestine (I−), we found the same quantity of ST16S rRNA copies, demonstrating the absence of interference when intestinal tissue was added. (PPTX) [file pone.0103069.s001.pptx]

## Slide 1
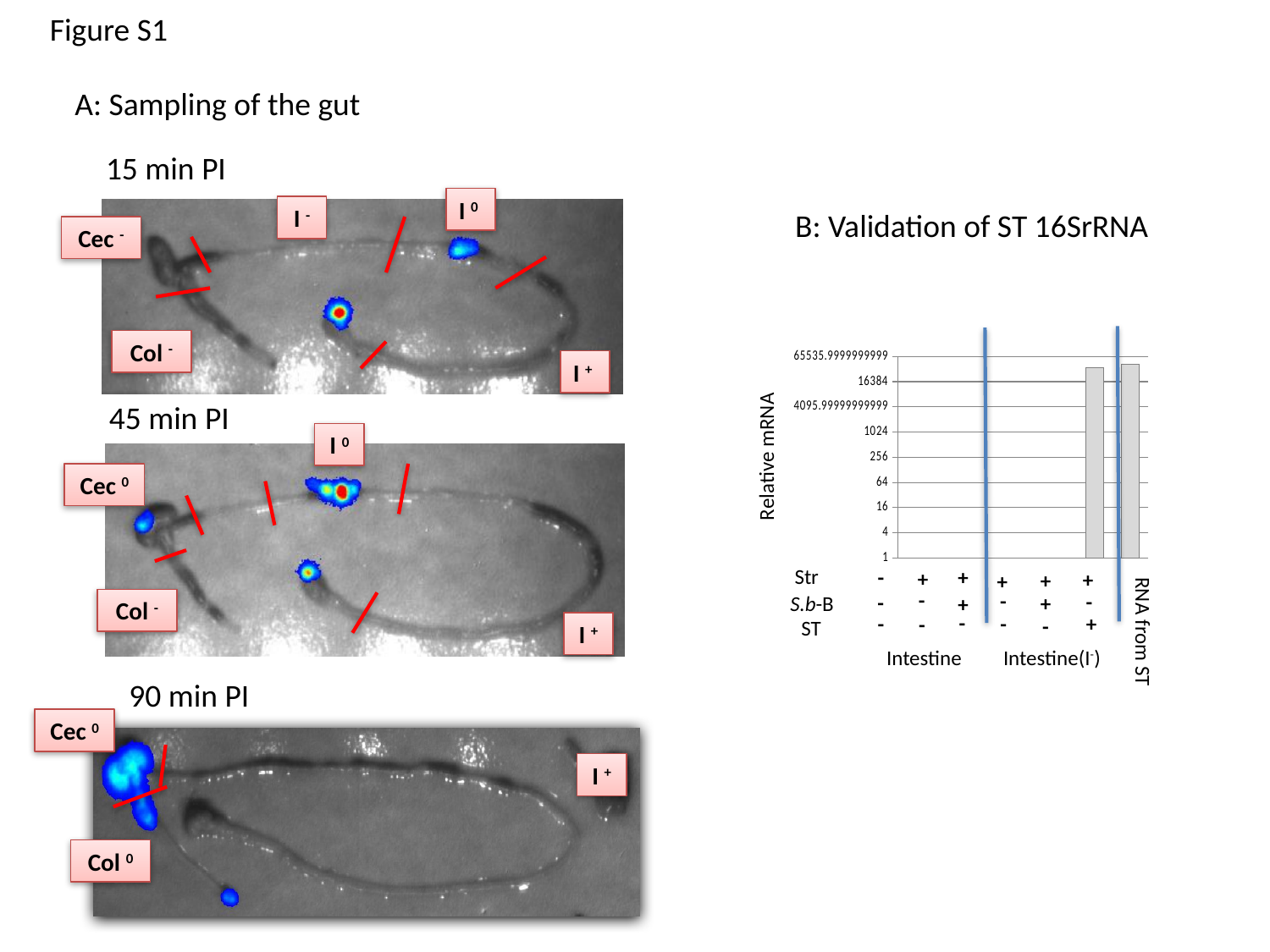

Figure S1
A: Sampling of the gut
15 min PI
I 0
I -
Cec -
Col -
I +
45 min PI
I 0
Cec 0
Col -
I +
90 min PI
Cec 0
I +
Col 0
B: Validation of ST 16SrRNA
### Chart
| Category | |
|---|---|
| T Norm_I_-_T1 | 1.0 |
| T Strepto_I_-_T2 | 0.0 |
| T Sb Strepto_I_-_T3 | 0.0 |
| WT_ I_-_1_15 min | 0.840138559342887 |
| WT_ I_-_1_15 min + Sb seules | 0.348275767731271 |
| WT_ I_-_1_15 min + WT seules | 35073.62083574408 |
| WT seules | 42027.97119311873 |Relative mRNA
Str
-
+
+
+
+
+
-
-
-
-
+
S.b-B
+
-
+
-
-
-
-
ST
RNA from ST
Intestine
Intestine(I-)
