# Supplementary material for: Saccharomyces boulardii Modifies Salmonella Typhimurium Traffic and Host Immune Responses along the Intestinal Tract
Source: PLoS One. 2014 Aug 13;9(8):e103069. doi: 10.1371/journal.pone.0103069 (PMC4145484; doi:10.1371/journal.pone.0103069)
Supplement: Figure S2 — In vivo imaging of the intestinal tract extracted 6 hours PI from control mice, mice treated by S.b -B alone, mice given ST- lux alone, and mice administered both S.b -B and ST- lux (A). Diagrams represent the intensity of light along the distance between the stomach and cecum. (B) Quantification of 16S rRNA in various parts of the GIT from mice infected by ST-lux alone and mice treated with S.b-B during infection. (PPTX) [file pone.0103069.s002.pptx]

## Slide 1
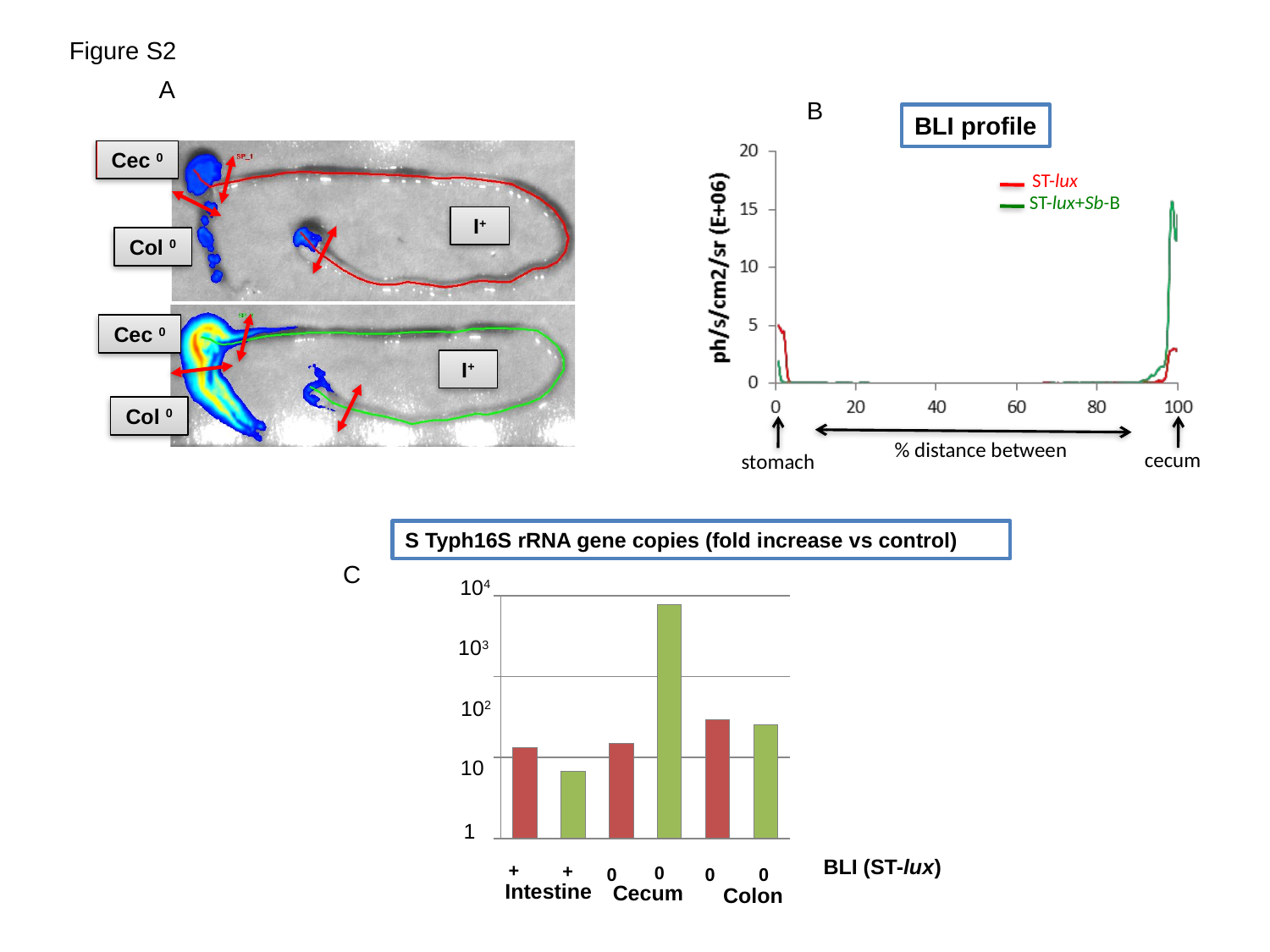

Figure S2
A
B
BLI profile
Cec 0
I+
Col 0
Cec 0
I+
Col 0
ST-lux
ST-lux+Sb-B
% distance between
cecum
stomach
S Typh16S rRNA gene copies (fold increase vs control)
C
104
### Chart
| Category | Typh w 36B4_I CEC Col_6h / T.Norm |
|---|---|
| WT_I_+_7_6h | 131.2917576297772 |
| WT+Sb_I_+_8_6h | 67.46783704281985 |
| WT_ CEC_O_7_6 h | 147.7839682246059 |
| WT+Sb_CEC_O_8_6 h | 7694.382141120468 |
| WT_ Col._O_7_6 h | 289.8669224477231 |
| WT+Sb_Col._O_8_6 h | 252.8143505205466 |103
102
10
1
BLI (ST-lux)
+
+
0
0
0
0
Intestine
Cecum
Colon
