# Supplementary material for: Saccharomyces boulardii Modifies Salmonella Typhimurium Traffic and Host Immune Responses along the Intestinal Tract
Source: PLoS One. 2014 Aug 13;9(8):e103069. doi: 10.1371/journal.pone.0103069 (PMC4145484; doi:10.1371/journal.pone.0103069)
Supplement: Figure S3 — IFN-γ and IL-10 gene expression measured by real-time PCR in the samples of intestine, cecum and colon from control mice (blue bars), and mice treated by streptomycin alone or with S.b -B. (PPTX) [file pone.0103069.s003.pptx]

## Slide 1
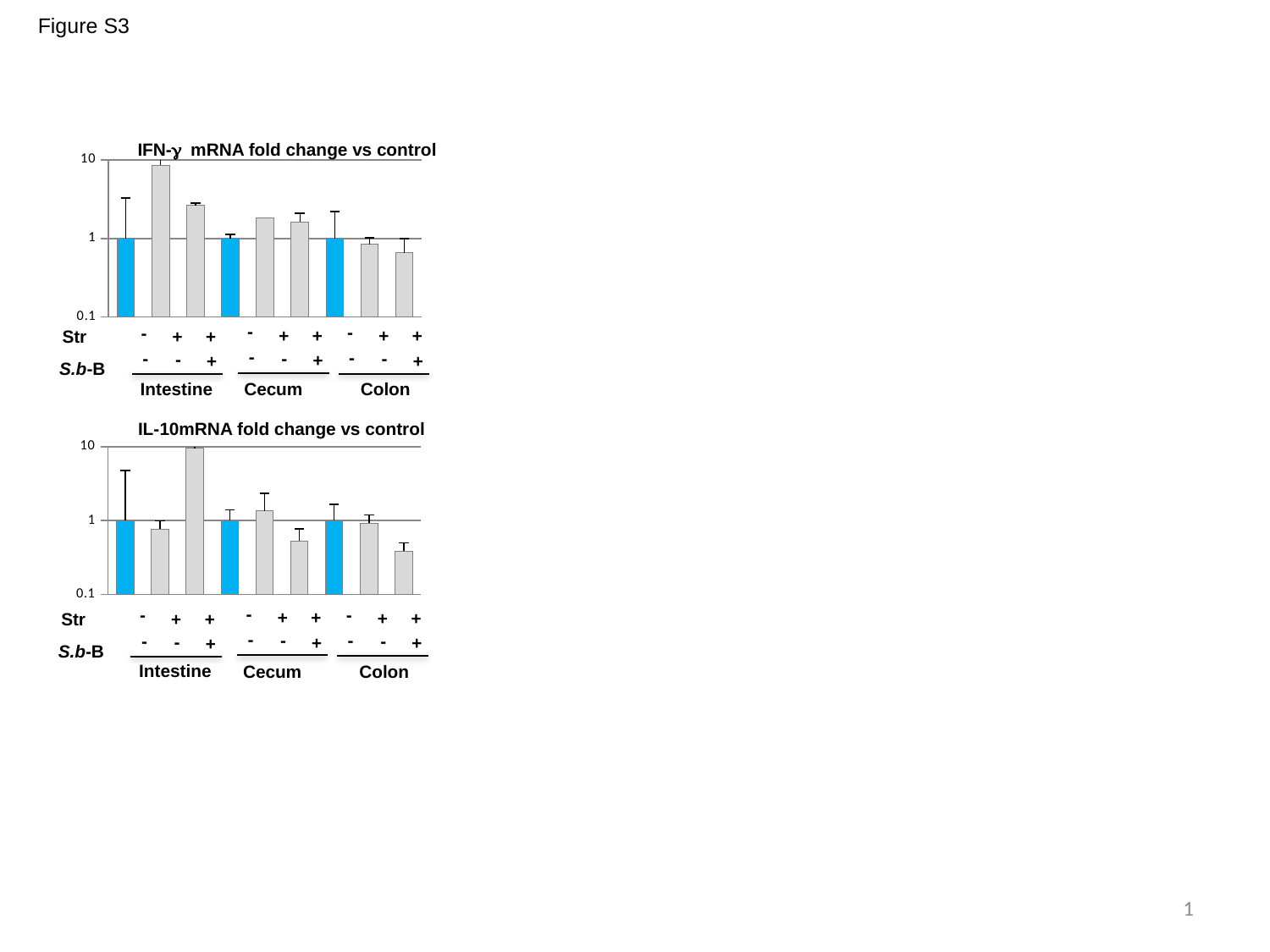

Figure S3
IFN-g mRNA fold change vs control
### Chart
| Category | IFN_Témoins I CEC Col / T.NORM |
|---|---|
| T Norm_I_-_T1_moy | 1.0 |
| T Strepto_I_-_T2_moy | 8.6038329563865 |
| T Sb Strepto_I_-_T3_moy | 2.649273396888606 |
| T Norm_CEC_-_T1_moy | 1.0 |
| T Strepto_CEC_-_T2_moy | 1.817000622939094 |
| T Sb Strepto_CEC_-_T3_moy | 1.616430511633212 |
| T Norm_Col._-_T1_moy | 1.0 |
| T Strepto_Col_-_T2_moy | 0.840907903174974 |
| T Sb Strepto_Col_-_T3_moy | 0.653382913734804 |-
+
+
-
-
+
-
+
+
-
-
+
-
+
+
-
-
+
Str
S.b-B
Intestine
Cecum
Colon
IL-10mRNA fold change vs control
### Chart
| Category | IL10_Témoins I CEC Col / T.NORM |
|---|---|
| T Norm_I_-_T1_moy | 1.0 |
| T Strepto_I_-_T2_moy | 0.765344899229321 |
| T Sb Strepto_I_-_T3_moy | 9.51445014900831 |
| T Norm_CEC_-_T1_moy | 1.0 |
| T Strepto_CEC_-_T2_moy | 1.343659407331773 |
| T Sb Strepto_CEC_-_T3_moy | 0.525912193897074 |
| T Norm_Col._-_T1_moy | 1.0 |
| T Strepto_Col_-_T2_moy | 0.924972857421226 |
| T Sb Strepto_Col_-_T3_moy | 0.382821104417269 |-
+
+
-
-
+
-
+
+
-
-
+
-
+
+
-
-
+
Str
S.b-B
Intestine
Cecum
Colon
1
