# Supplementary material for: Saccharomyces boulardii Modifies Salmonella Typhimurium Traffic and Host Immune Responses along the Intestinal Tract
Source: PLoS One. 2014 Aug 13;9(8):e103069. doi: 10.1371/journal.pone.0103069 (PMC4145484; doi:10.1371/journal.pone.0103069)
Supplement: Figure S4 — IFN-γ (A) and IL-10 (B) gene expression measured by real-time PCR in the different samples of intestine, cecum and colon obtained from mice infected by ST- lux alone and mice mice treated with S.b -B and infected for different periods of time (15, 45, 90 min and 6 hours). Empty bars: controls. PE: Photon Emission. Annotation of PE: “O” maximal PE, “−” no PE, “+” PE after ST passage. Data are representative of 5 independent experiments. (PPTX) [file pone.0103069.s004.pptx]

## Slide 1
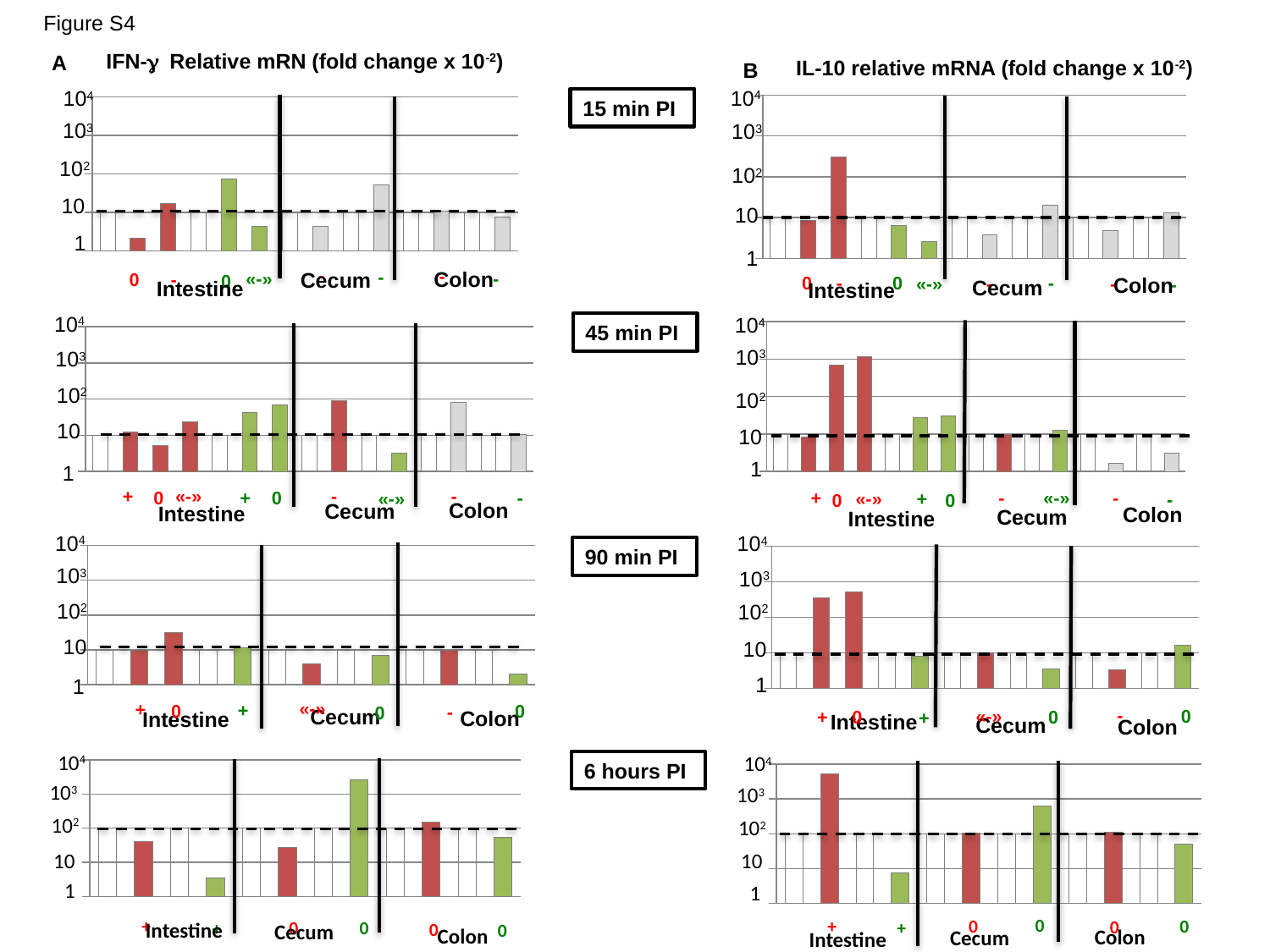

A
Figure S4
IFN-g Relative mRN (fold change x 10-2)
A
 IL-10 relative mRNA (fold change x 10-2)
B
### Chart
| Category | IL10_I CEC Col_15 min / T.STREPTO |
|---|---|
| T Strepto_I_-_T2 | 1.0 |
| WT_ I_O_1_15 min | 0.856886425896964 |
| WT_ I_-_1_15 min | 31.24169224394298 |
| T Sb Strepto_I_-_T3 | 1.0 |
| WT+Sb_I_O_2_15 min | 0.651645924871031 |
| WT+Sb_ I_-_2_15 min | 0.252903143706286 |
| T Strepto_CEC_-_T2 | 1.0 |
| WT_C_CEC_-_1_15 min | 0.378480136069454 |
| T Sb Strepto_CEC_-_T3 | 1.0 |
| WT+Sb_CEC_-_2_15 min | 2.056306436480002 |
| T Strepto_Col._-_T2 | 1.0 |
| WT_J_Col._-_1_15 min | 0.490779994067431 |
| T Sb Strepto_Col._-_T3 | 1.0 |
| WT+Sb_Col._-_2_15 min | 1.326781107315788 |104
### Chart
| Category | IFN_I CEC Col_15 min / T.STREPTO |
|---|---|
| T Strepto_I_-_T2 | 1.0 |
| WT_ I_O_1_15 min | 0.213188100993085 |
| WT_ I_-_1_15 min | 1.701932437909392 |
| T Sb Strepto_I_-_T3 | 1.0 |
| WT+Sb_I_O_2_15 min | 7.50907964175411 |
| WT+Sb_ I_-_2_15 min | 0.435063618270073 |
| T Strepto_CEC_-_T2 | 1.0 |
| WT_C_CEC_-_1_15 min | 0.431386266936483 |
| T Sb Strepto_CEC_-_T3 | 1.0 |
| WT+Sb_CEC_-_2_15 min | 5.090771029552652 |
| T Strepto_Col._-_T2 | 1.0 |
| WT_J_Col._-_1_15 min | 1.064088897116447 |
| T Sb Strepto_Col._-_T3 | 1.0 |
| WT+Sb_Col._-_2_15 min | 0.750662830358761 |104
15 min PI
103
103
102
102
10
10
1
1
-
-
-
-
«-»
-
0
0
Colon
Cecum
-
0
0
-
«-»
-
-
-
Colon
Cecum
Intestine
Intestine
104
### Chart
| Category | IL10_I CEC Col_45 min / T.STREPTO |
|---|---|
| T Strepto_I_-_T2 | 1.0 |
| WT_ I_+_3_45 min | 0.81932255330881 |
| WT_ I_O_3_45 min | 68.66184004510451 |
| WT_I_-_3_45 min | 115.7752167441341 |
| T Sb Strepto_I_-_T3 | 1.0 |
| WT+Sb_I_+_4bis_45 min | 2.683411765421788 |
| WT+Sb_I_O_4bis_45 min | 3.0474148435548 |
| T Strepto_CEC_-_T2 | 1.0 |
| WT_ CEC_-_3_45 min | 0.948509852708574 |
| T Sb Strepto_CEC_-_T3 | 1.0 |
| WT+Sb_CEC_O_4bis_45 min | 1.27641841342635 |
| T Strepto_Col._-_T2 | 1.0 |
| WT_moy Col._-_3_45 min | 0.162283137650668 |
| T Sb Strepto_Col._-_T3 | 1.0 |
| WT+Sb_Col._-_4bis_45 min | 0.307631148128157 |104
### Chart
| Category | IFN_I CEC Col_45 min / T.STREPTO |
|---|---|
| T Strepto_I_-_T2 | 1.0 |
| WT_ I_+_3_45 min | 1.242102468406321 |
| WT_ I_O_3_45 min | 0.512295289353457 |
| WT_I_-_3_45 min | 2.322824883060766 |
| T Sb Strepto_I_-_T3 | 1.0 |
| WT+Sb_I_+_4bis_45 min | 4.29054809669634 |
| WT+Sb_I_O_4bis_45 min | 6.994759305264285 |
| T Strepto_CEC_-_T2 | 1.0 |
| WT_ CEC_-_3_45 min | 9.001717864516682 |
| T Sb Strepto_CEC_-_T3 | 1.0 |
| WT+Sb_CEC_O_4bis_45 min | 0.318854948294443 |
| T Strepto_Col._-_T2 | 1.0 |
| WT_moy Col._-_3_45 min | 7.96677936084877 |
| T Sb Strepto_Col._-_T3 | 1.0 |
| WT+Sb_Col._-_4bis_45 min | 1.074584429609295 |45 min PI
103
103
102
102
10
10
1
1
+
«-»
-
-
0
0
+
+
-
-
-
«-»
«-»
«-»
+
-
0
0
Colon
Cecum
Intestine
Colon
Cecum
Intestine
### Chart
| Category | IFN_I CEC Col_90 min / T.STREPTO |
|---|---|
| T Strepto_I_-_T2 | 1.0 |
| WT_ I_+_5_90 min | 0.982286966011141 |
| WT_I_O_5_90 min | 3.133418573490228 |
| T Sb Strepto_I_-_T3 | 1.0 |
| WT+Sb_I_+_6_90 min | 1.152431816656662 |
| T Strepto_CEC_-_T2 | 1.0 |
| WT_ CEC_O_5_90 min | 0.395451179519219 |
| T Sb Strepto_CEC_-_T3 | 1.0 |
| WT+Sb_CEC_O_6_90 min | 0.701755965713592 |
| T Strepto_Col._-_T2 | 1.0 |
| WT_Col._-_5_90 min | 0.991822223999552 |
| T Sb Strepto_Col._-_T3 | 1.0 |
| WT+Sb_Col._O_6_90 min | 0.202280266615288 |104
104
### Chart
| Category | IL10_I CEC Col_90 min / T.STREPTO |
|---|---|
| T Strepto_I_-_T2 | 1.0 |
| WT_ I_+_5_90 min | 34.99525579228064 |
| WT_I_O_5_90 min | 50.85506987375089 |
| T Sb Strepto_I_-_T3 | 1.0 |
| WT+Sb_I_+_6_90 min | 0.803682796279063 |
| T Strepto_CEC_-_T2 | 1.0 |
| WT_ CEC_O_5_90 min | 1.023012350669598 |
| T Sb Strepto_CEC_-_T3 | 1.0 |
| WT+Sb_CEC_O_6_90 min | 0.357846590563899 |
| T Strepto_Col._-_T2 | 1.0 |
| WT_Col._-_5_90 min | 0.337726110808512 |
| T Sb Strepto_Col._-_T3 | 1.0 |
| WT+Sb_Col._O_6_90 min | 1.656620187110668 |90 min PI
103
103
102
102
10
10
1
1
«-»
+
+
0
0
-
0
-
0
«-»
0
+
0
+
Cecum
Colon
Intestine
Intestine
Cecum
Colon
### Chart
| Category | IFN_I CEC Col_6h / T.STREPTO |
|---|---|
| T Strepto_I_-_T2 | 1.0 |
| WT_I_+_7_6h | 0.405033624712916 |
| T Sb Strepto_I_-_T3 | 1.0 |
| WT+Sb_I_+_8_6h | 0.0343732073068134 |
| T Strepto_CEC_-_T2 | 1.0 |
| WT_ CEC_O_7_6 h | 0.278065331350794 |
| T Sb Strepto_CEC_-_T3 | 1.0 |
| WT+Sb_CEC_O_8_6 h | 26.60913164397843 |
| T Strepto_Col._-_T2 | 1.0 |
| WT_ Col._O_7_6 h | 1.499296518763308 |
| T Sb Strepto_Col._-_T3 | 1.0 |
| WT+Sb_Col._O_8_6 h | 0.530122016045276 |104
103
102
10
1
+
0
0
+
0
0
Intestine
Cecum
Colon
104
### Chart
| Category | IL10_I CEC Col_6h / T.STREPTO |
|---|---|
| T Strepto_I_-_T2 | 1.0 |
| WT_I_+_7_6h | 53.78593123752336 |
| T Sb Strepto_I_-_T3 | 1.0 |
| WT+Sb_I_+_8_6h | 0.0742027165154381 |
| T Strepto_CEC_-_T2 | 1.0 |
| WT_ CEC_O_7_6 h | 1.067188370242186 |
| T Sb Strepto_CEC_-_T3 | 1.0 |
| WT+Sb_CEC_O_8_6 h | 6.116289069578841 |
| T Strepto_Col._-_T2 | 1.0 |
| WT_ Col._O_7_6 h | 1.103651277426838 |
| T Sb Strepto_Col._-_T3 | 1.0 |
| WT+Sb_Col._O_8_6 h | 0.509753642940216 |103
102
10
1
0
+
0
0
0
+
Colon
Cecum
Intestine
6 hours PI
