# Supplementary material for: Saccharomyces boulardii Modifies Salmonella Typhimurium Traffic and Host Immune Responses along the Intestinal Tract
Source: PLoS One. 2014 Aug 13;9(8):e103069. doi: 10.1371/journal.pone.0103069 (PMC4145484; doi:10.1371/journal.pone.0103069)
Supplement: Figure S5 — GM-CSF, IL-1 and TNF-α gene expression measured by real-time PCR 6 hours PI in the cecum from mice infected by ST- lux alone and infected mice treated with S.b -B. (PPTX) [file pone.0103069.s005.pptx]

## Slide 1
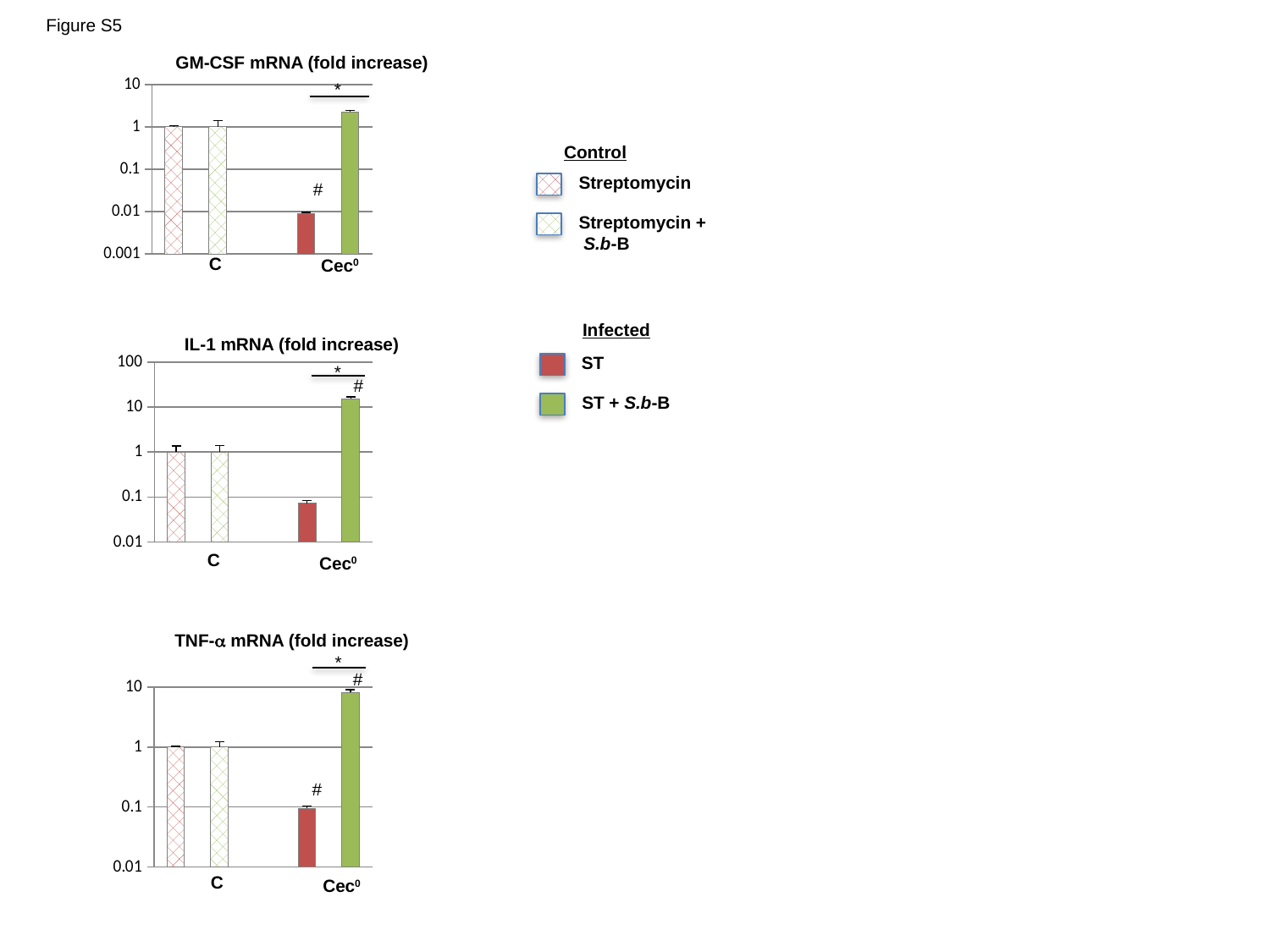

Figure S5
GM-CSF mRNA (fold increase)
### Chart
| Category | |
|---|---|
| T Strepto_I_-_T2_moy | 1.0 |
| T Sb Strepto_I_-_T3_moy | 1.0 |
| | None |
| ST_O_6h | 0.00893789415247845 |
| STSb_O_6h | 2.278803622515861 |*
#
Control
Streptomycin
Streptomycin +
 S.b-B
C
Cec0
Infected
ST
ST + S.b-B
### Chart
| Category | |
|---|---|
| T Strepto_I_-_T2_moy | 1.0 |
| T Sb Strepto_I_-_T3_moy | 1.0 |
| | None |
| ST_O_6h | 0.0714444738630955 |
| STSb_O_6h | 15.14466571568022 |IL-1 mRNA (fold increase)
*
#
C
Cec0
TNF-a mRNA (fold increase)
*
### Chart
| Category | |
|---|---|
| T Strepto_I_-_T2_moy | 1.0 |
| T Sb Strepto_I_-_T3_moy | 1.0 |
| | None |
| ST_O_6h | 0.0955340253572845 |
| STSb_O_6h | 8.159246193053523 |#
#
C
Cec0
