# Supplementary figures and images for: Saccharomyces boulardii Modifies Salmonella Typhimurium Traffic and Host Immune Responses along the Intestinal Tract
Source: PLoS One. 2014 Aug 13;9(8):e103069. doi: 10.1371/journal.pone.0103069 (PMC4145484; doi:10.1371/journal.pone.0103069)

## Slide 1
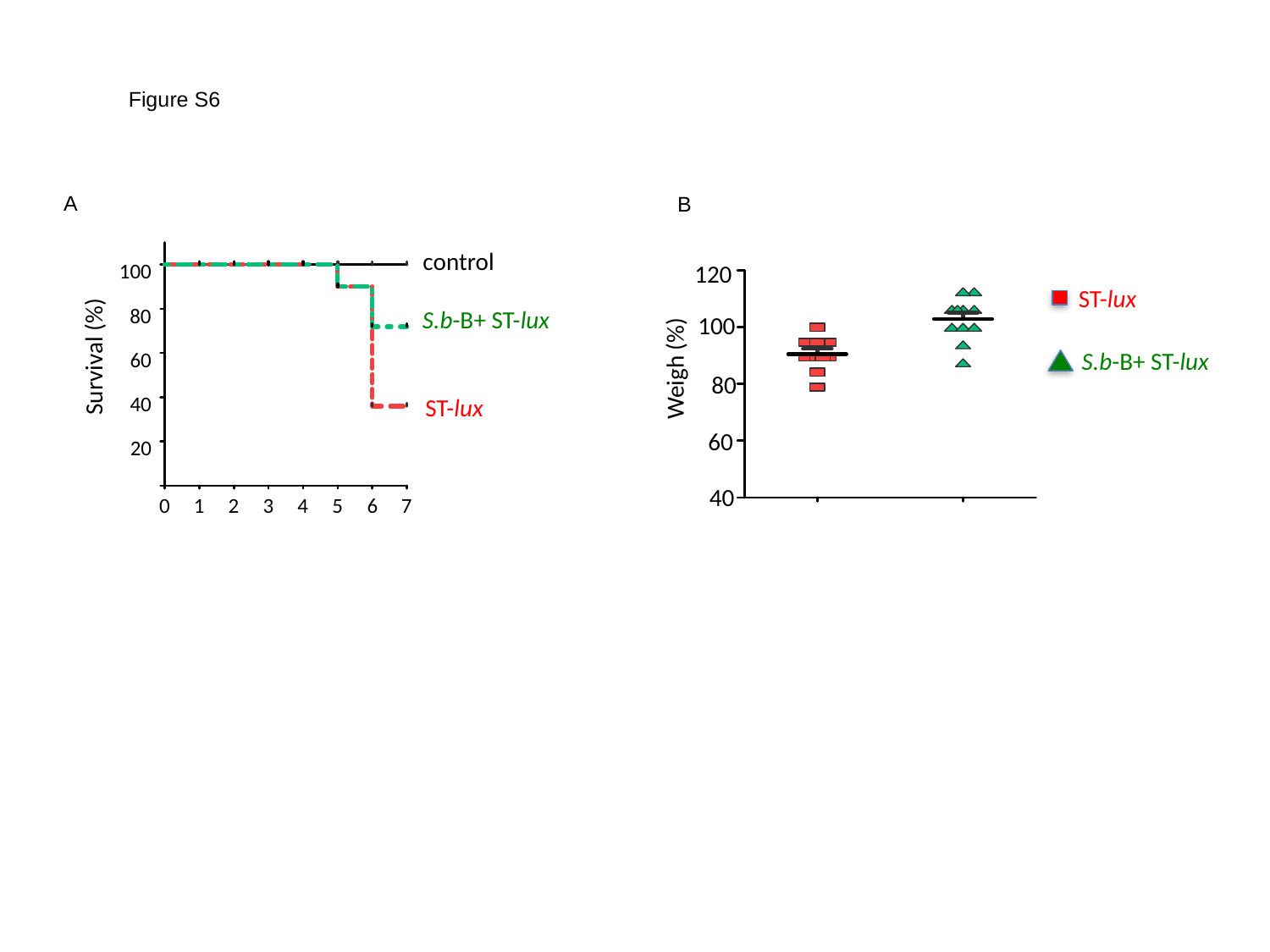

Figure S6
A
B
ST-lux
Weigh (%)
S.b-B+ ST-lux
120
100
80
60
40
100
80
60
40
20
6
4
1
2
3
5
0
7
control
S.b-B+ ST-lux
Survival (%)
ST-lux

Supplement: Figure S6 — S.b -B treatment inhibits wasting disease due to ST-infection. Survival was monitored every day up until 6 days PI (A). Weight loss on day 5 PI is expressed as a % compared to the initial weight of the mice before infection (B). (PPTX) [file pone.0103069.s006.pptx]
