# Supplementary material for: Saccharomyces boulardii Modifies Salmonella Typhimurium Traffic and Host Immune Responses along the Intestinal Tract
Source: PLoS One. 2014 Aug 13;9(8):e103069. doi: 10.1371/journal.pone.0103069 (PMC4145484; doi:10.1371/journal.pone.0103069)
Supplement: Figure S7 — Experimental protocol. (PPTX) [file pone.0103069.s007.pptx]

## Slide 1
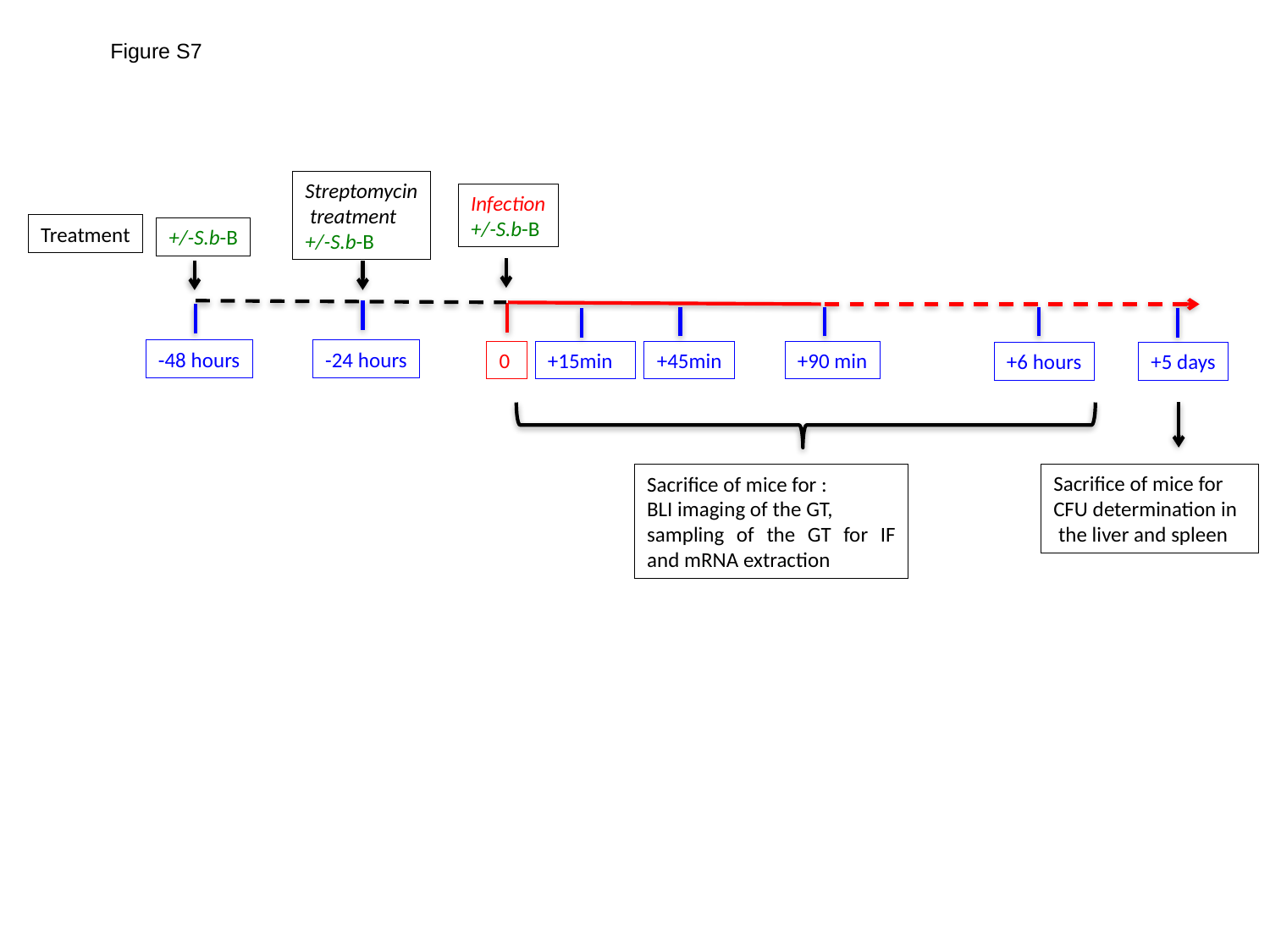

Figure S7
Streptomycin
 treatment
+/-S.b-B
Infection
+/-S.b-B
Treatment
+/-S.b-B
-48 hours
-24 hours
+90 min
+15min
+45min
0
+5 days
+6 hours
Sacrifice of mice for
CFU determination in
 the liver and spleen
Sacrifice of mice for :
BLI imaging of the GT,
sampling of the GT for IF and mRNA extraction
