# Supplementary material for: Saccharomyces boulardii Modifies Salmonella Typhimurium Traffic and Host Immune Responses along the Intestinal Tract
Source: PLoS One. 2014 Aug 13;9(8):e103069. doi: 10.1371/journal.pone.0103069 (PMC4145484; doi:10.1371/journal.pone.0103069)
Supplement: Table S1 — Mean concentration (CFU×106/g of tissue) of Salmonella detected along the intestinal tract after oral inoculation of mice. Different portions of the intestinal tract were removed, weighed and homogenized for plating of serial dilutions onto McConkey agar plates, as described in Material and Methods. Data are expressed as CFU×106/g of tissue. ND: not determined. N = 5. (DOCX) [file pone.0103069.s008.docx]

Table S1

|  | Time after Salmonella (ST-lux) and *S. b*-B challenge | | | | | |
| --- | --- | --- | --- | --- | --- | --- |
|  | 15 min | | 45 min | | 90 min | |
|  | ST-*lux* | ST-*lux*  + *S.b*-B | ST-*lux* | ST-*lux*  + *S.b*-B | ST-*lux* | ST-*lux*  + *S.b*-B |
| Duodenum | 6 ± 0.1 | 10 ± 1.1 | 2.6 ± 0.4 | 1.6 ± 0.2 | 0 | 0 |
| Jejunum | 50 ± 4.5 | 80 ± 8.5 | 150 ± 22 | 10 ± 1.4 | ND | ND |
| Ileum | 3.6 ± 0.4 | 5 ± 0.8 | 3 ± 0.35 | 0 | 3 ± 0.4 | 60 ± 7 |
| Cecum | 0 | 0 | 0.06 ±0.005 | 0.03 ±0.002 | 70 ± 7.8 | 12 ± 1.2 |
