# Supplementary material for: Saccharomyces boulardii Modifies Salmonella Typhimurium Traffic and Host Immune Responses along the Intestinal Tract
Source: PLoS One. 2014 Aug 13;9(8):e103069. doi: 10.1371/journal.pone.0103069 (PMC4145484; doi:10.1371/journal.pone.0103069)
Supplement: Table S2 — Mean concentration (CFU×105/g of tissue) of S.b -B detected along the intestinal tract after oral inoculation of mice. Different portions of the intestinal tract were removed, weighed and homogenized for plating of serial dilutions onto YEPD agar plates, as described in Material and Methods. Data are expressed as CFU×105/g of tissue. ND: not determined. N = 5. (DOCX) [file pone.0103069.s009.docx]

Table S2

|  | Time after Salmonella and *S.b* -B challenge | | | |
| --- | --- | --- | --- | --- |
|  | 15 min | 45 min | 90 min | 6 hours |
| Duodenum | 10 ± 0.09 | 0 | 0 | ND |
| Jejunum | 50 ± 4 | 4.2 ± 0.5 | ND | ND |
| Ileum | 0 | 0 | 2.6 ± 0.3 | ND |
| Cecum | 0 | 0 | 0.3 ± 0.02 | 1 ± 0.05 |
