# Supplementary material for: Saccharomyces boulardii Modifies Salmonella Typhimurium Traffic and Host Immune Responses along the Intestinal Tract
Source: PLoS One. 2014 Aug 13;9(8):e103069. doi: 10.1371/journal.pone.0103069 (PMC4145484; doi:10.1371/journal.pone.0103069)
Supplement: Table S3 — Primers used in this study. (DOC) [file pone.0103069.s010.doc]

Table S3

| Salmonella | **Typh** | **Fw** | TGTTGTGGTTAATAACCGCA |
| --- | --- | --- | --- |
| Salmonella | **Typh** | **Rev** | GACTACCAGGGTATCTAATCC |
| Mouse | **36B4** | **Fw** | TCCAGGCTTTGGGCATCA |
| Mouse | **36B4** | **Rev** | CTTTATCAGCTGCACATCACTCAGA |
| Mouse | **GM-CSF** | **Fw** | GCCACAGTTGGAAGGCAGTA |
| Mouse | **GM-CSF** | **Rev** | AAATATAATGGTCCCTATCAGTAGAAA |
| Mouse | **IFN-γ** | **Fw** | CAGCAACAGCAAGGCGAAA |
| Mouse | **IFN-γ** | **Rev** | CTGGACCTGTGGGTTGTTGAC |
| Mouse | **IL-1β** | **Fw** | TCGCTCAGGGTCACAAGAAA |
| Mouse | **IL-1β** | **Rev** | CATCAGAGGCAAGGAGGAAAAC |
| Mouse | **IL-10** | **Fw** | GGTTGCCAAGCCTTATCGGA |
| Mouse | **IL-10** | **Rev** | ACCTGCTCCACTGCCTTGCT |
| Mouse | **IL-4** | **Fw** | TCTCGAATGTACCAGGAGCCATA |
| Mouse | **IL-4** | **Rev** | CGTTCAAAATGCCGATGATCT |
| Mouse | **TNF- α** | **Fw** | AGGCTGCCCCGACTACGT |
| Mouse | **TNF- α** | **Rev** | GACTTTCTCCTGGTATGAGATAGCAAA |
